# Activity Recognition for Persons With Stroke Using Mobile Phone Technology: Toward Improved Performance in a Home Setting

**DOI:** 10.2196/jmir.7385

**Published:** 2017-05-25

**Authors:** Megan K O'Brien, Nicholas Shawen, Chaithanya K Mummidisetty, Saninder Kaur, Xiao Bo, Christian Poellabauer, Konrad Kording, Arun Jayaraman

**Affiliations:** ^1^ Max Nader Lab for Rehabilitation Technologies and Outcomes Research Rehabilitation Institute of Chicago Chicago, IL United States; ^2^ Department of Physical Medicine and Rehabilitation Northwestern University Chicago, IL United States; ^3^ Department of Computer Science and Engineering University of Notre Dame Notre Dame, IN United States

**Keywords:** smartphone, activities of daily living, ambulatory monitoring, machine learning, stroke rehabilitation

## Abstract

**Background:**

Smartphones contain sensors that measure movement-related data, making them promising tools for monitoring physical activity after a stroke. Activity recognition (AR) systems are typically trained on movement data from healthy individuals collected in a laboratory setting. However, movement patterns change after a stroke (eg, gait impairment), and activities may be performed differently at home than in a lab. Thus, it is important to validate AR for gait-impaired stroke patients in a home setting for accurate clinical predictions.

**Objective:**

In this study, we sought to evaluate AR performance in a home setting for individuals who had suffered a stroke, by using different sets of training activities. Specifically, we compared AR performance for persons with stroke while varying the origin of training data, based on either population (healthy persons or persons with stoke) or environment (laboratory or home setting).

**Methods:**

Thirty individuals with stroke and fifteen healthy subjects performed a series of mobility-related activities, either in a laboratory or at home, while wearing a smartphone. A custom-built app collected signals from the phone’s accelerometer, gyroscope, and barometer sensors, and subjects self-labeled the mobility activities. We trained a random forest AR model using either healthy or stroke activity data. Primary measures of AR performance were (1) the mean recall of activities and (2) the misclassification of stationary and ambulatory activities.

**Results:**

A classifier trained on stroke activity data performed better than one trained on healthy activity data, improving average recall from 53% to 75%. The healthy-trained classifier performance declined with gait impairment severity, more often misclassifying ambulatory activities as stationary ones. The classifier trained on in-lab activities had a lower average recall for at-home activities (56%) than for in-lab activities collected on a different day (77%).

**Conclusions:**

Stroke-based training data is needed for high quality AR among gait-impaired individuals with stroke. Additionally, AR systems for home and community monitoring would likely benefit from including at-home activities in the training data.

## Introduction

Recovering independent mobility after a stroke, both at home and in the community, is a priority for most stroke survivors [1]. The development of therapeutic interventions to restore walking and functional recovery remains a major focus of rehabilitation for individuals after stroke [2]. However, it is difficult to know which rehabilitation strategies most improve functional mobility. As stroke units strive to optimize inpatient and outpatient care and shorten hospital stays, it becomes increasingly important to measure the impact of rehabilitation beyond the traditional clinical setting.

Monitoring daily physical activity is essential to understanding patient recovery. After all, community-dwelling stroke patients exhibit high levels of sedentary behavior [3,4], which has been identified as a risk factor for secondary cardiovascular disease and mortality [5]. Decreasing sedentary time by increasing ambulation or even standing time may reduce these risks [6,7] and prevent further health complications for persons with stroke. Wearable sensors, coupled with activity recognition (AR) models and machine learning techniques, can identify various mobility-related activities, such as sitting, lying, standing, walking, and stair use, in the clinic and in the home and community. This enables therapists to develop personalized, data-driven programs to advise patients and improve activity levels [8]. Thus, efficiently measuring mobility activities through a wearable AR system is a major quantitative outcome measure for studying new therapeutic interventions for stroke survivors.

Smartphones in particular have proven promising for unobtrusive health monitoring among patients [9], as they are now inexpensive, widely used, have an integrated system of movement-related sensors, and are able to transmit data continuously. Additionally, with access to the Global Positioning System (GPS), smartphones can allow clinicians and researchers to quantify outdoor community mobility [10-12] for tracking recovery and societal reintegration. Smartphones offer a favorable alternative to current monitoring devices such as pedometers, step activity monitors (SAMs), or other accelerometry-driven products [13], which focus exclusively on step counts and walking bouts.

A significant obstacle to the deployment of AR systems in rehabilitation is their reliability when applied to patients. Recent work has demonstrated that AR classifiers trained using in-lab data from young, able-bodied adults do not generalize to older adults [14], persons with Parkinson’s disease [15], or patients with lower limb impairments [16]. Rather, using training data from the neurological population of interest notably improved AR accuracy, likely because such groups exhibit different movement patterns than a young, healthy cohort [17]. Despite some ongoing research for stroke-based AR [18], we know little about the generalization of AR classifiers from healthy subjects to stroke patients. In particular, the heterogeneous movement-related outcomes that accompany stroke, such as level of gait impairment, may affect AR performance. It remains to be seen whether activities from a healthy cohort provide sufficient training data to classify stroke activities.

Another obstacle for the deployment of AR systems in rehabilitation is their reliability when applied to at-home activities. That is, AR classifier training usually relies on activities performed in a laboratory, but the end goal for these classifiers is to detect activities performed elsewhere in the community. Considering that a laboratory is an unfamiliar environment under close researcher supervision, it stands to reason that activities performed in a lab may look different from those performed at home. It is thus critical to know whether in-lab activities provide sufficient training data to classify at-home activities or whether models should be tuned to the environment of interest.

Here, we set out to investigate AR for individuals with stroke—specifically, the dependence of AR performance on the type of training data (from stroke or healthy subjects) and the environment (laboratory or home setting). Young, healthy individuals and community ambulators with stroke wore a smartphone while performing and self-labeling various mobility-related activities. We compared the ability of AR models trained on either a healthy or stroke cohort to classify activities in stroke cohorts with different levels of gait impairment. We also compared the ability of AR models trained on activities collected in either a laboratory or a home setting to classify activities at home for a stroke cohort. This approach is an important first step in highlighting potential issues with home monitoring using traditional laboratory-based AR methods.

## Methods

### Participants

A total of 30 individuals with stroke (mean 60.7, SD 13.3 years; 18 males and 12 females) participated in this study. Of these subjects, 21 had ischemic strokes, and 9 had hemorrhagic strokes; 16 sustained right-side damage, and 14 sustained left-side damage. Median stroke latency was 4.6 years (range 1986-2015) at the time of the study. Exclusion criteria included severe cognitive impairment (scoring ≤17 points on the Mini–Mental State Examination [19] and physical impairment that would inhibit ability to use a smartphone. We determined gait impairment using preferred walking speed during a 10-meter walk test (10MWT). This method categorized 8 subjects with mild impairment (>0.8 m/s), 13 subjects with moderate impairment (≥0.4 and ≤0.8 m/s), and 9 subjects with severe impairment (<0.4 m/s). Fifteen healthy subjects (mean 31.1, SD 9.2 years; 4 males and 11 females) were also recruited for this study, using a sample of convenience.

All subjects provided written informed consent before participation. The study was approved by the Institutional Review Board of Northwestern University (Chicago, IL) in accordance with federal regulations, university policies, and ethical standards regarding research on human subjects.

### Smartphone Sensing System

Subjects wore a Samsung Galaxy S4 running Android OS 4.4.4 on their waist in a belted pouch. The pouch was not restricted to any particular location on their waists (eg, right or left side) to make the home deployment as realistic as possible. A custom app named CIMON [20,21] collected tri-axial accelerometer data at an average of 60 samples per second, gyroscope data at 60 samples per second, and barometer data at 6 samples per second. Subjects self-labeled various mobility-related activities through CIMON’s user interface. Sensor data and labels were sent in real time via WiFi and LTE to a HIPAA-compliant (Health Insurance Portability and Accountability Act of 1996), secure server at the University of Notre Dame (Notre Dame, IN).

### Activity Labeling

Subjects with stroke performed and labeled a sequence of six activities (Sitting, Lying, Standing, Stairs Up, Stairs Down, and Walking) during two in-lab sessions, and they performed another session independently at home. During their first visit to the lab, they completed the activity sequence three times between rest periods. They were then asked to perform each of the six activities at least twice at home on the following day. They returned to the lab on the third day to complete three additional sequences. The healthy subjects performed and labeled the same activities at their leisure over a two-week period, and we asked that they complete each activity at least twice per day. Before taking the phones home, all subjects were taught to use the app and labeling system, and they performed several activities under the supervision of the researchers to ensure understanding.

Labeling an activity involved removing the phone from its pouch, selecting an activity label from a dropdown menu on the user interface, replacing the phone in the pouch, and commencing with the activity. This approach generated noisy, high-frequency sensor signals unrelated to the movement of interest when removing and replacing the phone. We removed these extraneous signals using an activity-dependent threshold on sample entropy, supplemented by manual trimming, at the beginning and end of each label. We also removed any trials that the subjects had obviously mislabeled, which occasionally occurred in the Home sessions (eg, a brief “Walking” trial with nearly flat signals from the accelerometer and gyroscope sensors, suggesting that the subject selected the incorrect label and neglected to cancel the trial).

### Activity Recognition

Data processing, activity recognition, and model analysis were implemented in MATLAB 2016b (MathWorks; Natick, MA). Accelerometer and gyroscope data were resampled to 50 Hz, and barometer data were resampled to 6 Hz to correct for any variability in the sensors’ sampling frequencies. Each activity recording was then divided into 10-second data clips (instances) with 90% overlap.

Initially, 270 features were identified from the activity data clips. Of these features, 131 arose from the accelerometer, 131 arose from the gyroscope, and 8 arose from the barometer. Features included statistical measures of the sensor signal, its derivatives, and the frequency domain (Tables 1 and 2).

To reduce the complexity of the model, a reduced feature set was chosen using MATLAB’s Out-of-Bag Predictor Importance method for a random undersampling (RUS) random forest (RF) model trained on the healthy subject data. RF models are often used in AR for their efficiency and flexibility [22,23], maintaining high accuracy for multiclass problems and large feature sets. The final feature set included 151 features, with 80 from the accelerometer, 63 from the gyroscope, and 8 from the barometer.

**Table 1:** Activity recognition model features from accelerometer and gyroscope signals.

Description	Number of features
Mean, range, and interquartile range	3 (per axis)
Moments: standard deviation, skew, and kurtosis	3 (per axis)
Histogram: bin counts of z-scores from -2 to 2	4 (per axis)
Moments of derivative: mean, standard deviation (SD), skew, and kurtosis	4 (per axis)
Mean of the squared norm	1
Sum of axial standard deviations	1
Pearson correlation coefficient, r(xy), r(xz), r(yz)	1 (per axis)
Mean cross products (raw and normalized), xy, xz, yz	2 (per axis)
Absolute mean of cross products (raw and normalized)	2 (per axis)
Power spectra: mean, standard deviation, skew, and kurtosis	4 (per axis)
Mean power in 0.5 Hz bins from 0-10 Hz	20 (per axis)

**Table 2:** Activity recognition model features from barometer signals.

Description	Number of features
Moments of derivative: mean, SD, skew, and kurtosis	4
SD, range, and interquartile range	3
Slope of linear regression	1

The same analysis was also performed using the stroke subject data, resulting in a set of 112 features, of which 102 were shared with the set of 151 features from the healthy subject data. Because of this similarity, we opted to use the set of 151 features throughout our models for simplicity. The chance of overfitting to the Healthy training set was negligible since less than half of the available features were discarded and the set overlapped substantially with features derived from the Stroke training set. Further potential for overfitting could have been removed if features were selected based on an external set not used for model training or testing, or by using an additional layer of cross-validation to the same effect.

RF tends to misclassify activities that are underrepresented in the data and can underperform on the Stairs Up and Stairs Down activities because of their rarity within the training data (respectively accounting for 1.68% (4138/246,283) and 1.51% (3723/246,823) of feature vectors). We implemented the RUSBoost algorithm [24] using decision trees (minimum leaf size=5, learn rate=1, number of trees=200) to improve model performance for an imbalanced class distribution (eg, underrepresented stair activity, overrepresented sitting activity). This number of trees was found to be sufficient for nearly full learning without overfitting. Using RUSBoost increased mean recall of Stairs Up by 6.5% and Stairs Down by 3.3% over an RF model, while showing little average change in recall over the remaining activities. Based on this finding, we used RUSBoost in the remainder of our analysis.

### Model Analysis

**Figure 1:**
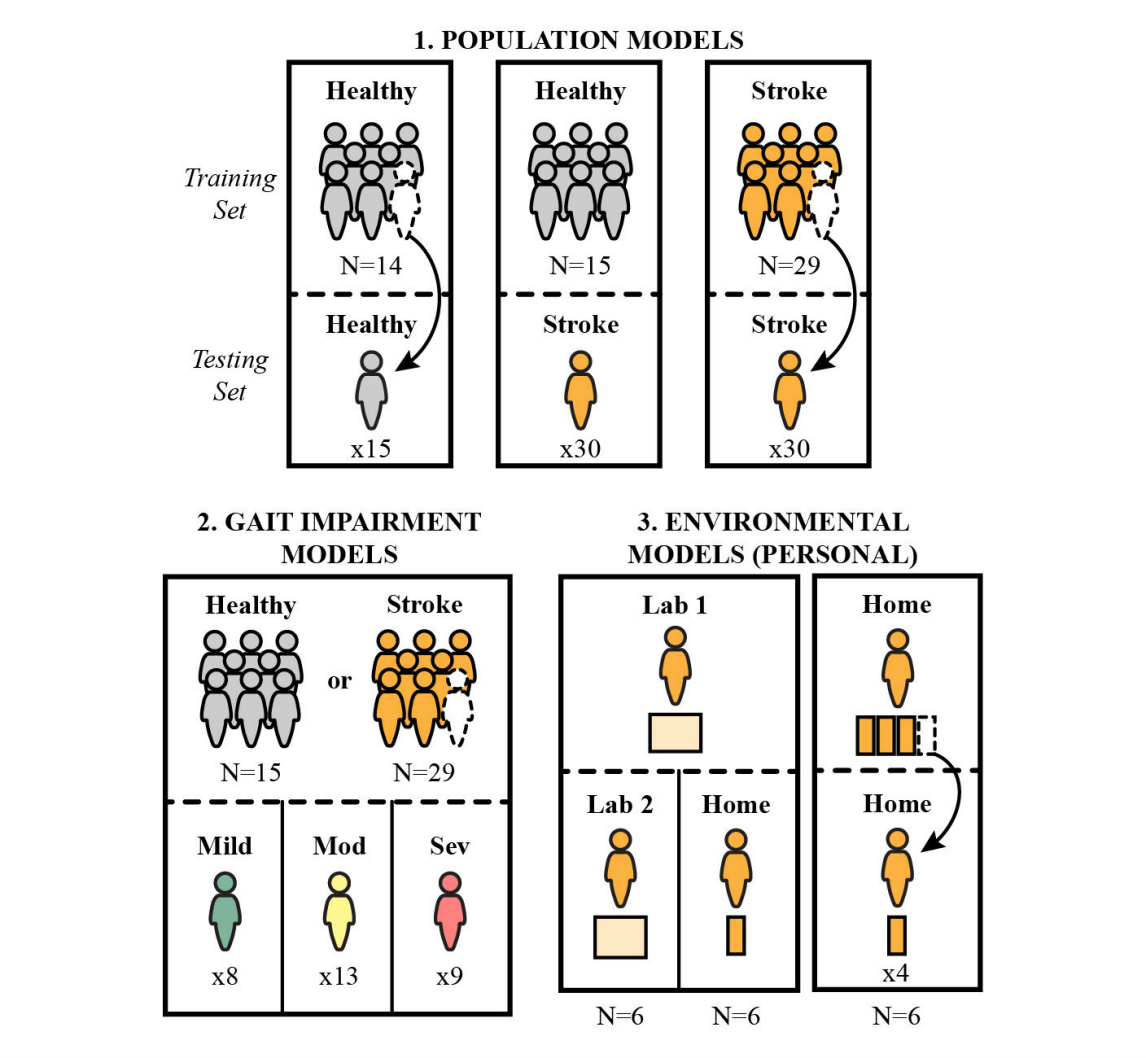
Efficacy of stroke AR was compared using three types of models.

We evaluated various AR classifiers for their performance in stroke activity recognition. We designated these classifiers as follows: (1) population models comparing the performance of a model trained using activity data from young, healthy subjects (Healthy) versus older stroke subjects (Stroke); (2) gait impairment models comparing the performance of a young, healthy training set on stroke subjects with mild versus moderate versus severe gait impairment; and (3) environmental models for stroke subjects, comparing the ability of the first in-lab training set (Lab 1) to recognize at-home activities (Home) versus the in-lab activities performed on a separate day (Lab 2). We also designed an environmental model trained and tested on only at-home activities. These model comparisons and their respective training and testing sets are depicted in Figure 1.

Population models were trained and tested on either the Healthy or Stroke subject data using leave-one-subject-out cross validation (Healthy-to-Healthy, Stroke-to-Stroke). The performance of the Healthy population model, trained on all 15 healthy subjects, was also evaluated for each Stroke subject (Healthy-to-Stroke). The Healthy-to-Healthy model yielded a baseline AR performance, against which the other models were compared.

To further probe the efficacy of Healthy data in detecting activities for persons with stroke, the Healthy-to-Stroke model results were separated for different subgroups of the stroke population based on gait impairment (Mild, Moderate, and Severe). We hypothesized that AR models trained on Healthy activities would perform better for subjects with less gait impairment (ie, a Healthy-to-Mild model would perform better than a Healthy-to-Severe model) and that this improved performance would be more pronounced for ambulatory activities (Stairs Up, Stairs Down, and Walking). We performed a similar analysis using the Stroke population training data to determine whether the performance of the Stroke population model is affected by level of gait impairment. We hypothesized that the mixed-impairment Stroke population model would perform similarly across impairment subgroups.

Environmental models assessed the capacity of training data collected in a laboratory setting to classify activities performed at home. We used personal models trained on Lab 1 data and tested on Lab 2 data for a baseline comparison (Lab-1-to-Lab 2), as well as Home data (Lab 1-to-Home). For the same set of subjects, we designed personal models trained on Home data and tested on other Home data via a four-fold cross validation (Home-to-Home). In this model, the Home data were divided into four folds chronologically rather than randomly, selected to ensure no overlap between adjacent folds. We used only subjects who had at least 60 seconds of each activity in each environmental setting (Lab 1, Home, and Lab 2), which limited the pool to six subjects. With this smaller dataset, we reduced the number of classes to four (combining Sitting and Lying as well as Stairs Up and Stairs Down) to focus on the broader misclassification of stationary and ambulatory activities. We hypothesized that the Lab 1-to-Lab 2 model and the Home-to-Home model would perform better than the Lab 1-to-Home model, expecting differences to arise in subject behavior and in the relative distribution of activities when performing activities at home versus in the lab.

We focused on personal models for the most direct comparison of AR efficacy in the Lab and Home environments. In a practical implementation of AR, a global model—with a training set based on multiple subjects—would be used to classify activities from a new subject, as in the population models described above. In order to represent this use-case scenario, we also examined global models for the three environmental analyses: Lab 1-to-Lab 2, Lab 1-to-Home, and Home-to-Home. The environmental global models were implemented using leave-one-subject-out cross validation on the same group of six subjects evaluated in the personal models.

#### Barometer Sensor Validation

The barometer is a relatively new feature in smartphone technology, and it is important to determine whether this sensor’s signals provide useful information to AR algorithms. Much of AR to date has relied predominantly on movement signals from accelerometers and, more recently, gyroscopes to distinguish between daily mobility-related activities. We thus examined the Healthy-to-Healthy model with and without the 8 barometer features to assess whether this sensor contributes to overall AR performance.

We found that the barometer improved recognition of stairs activity, similar to the findings of Del Rosario et al [14]. Specifically, including the 8 barometer features reduced misclassification of Stairs Up as Walking (from a misclassification of 31.32% [1296/4138] to 13.12% [543/4238]) and reduced misclassification of Stairs Down as Walking (from a misclassification of 33.04% [1230/3723] to 14.07% [524/3723]). Including these features also decreased misclassification of Stairs Down as Stairs Up (from 17.67% [731/4138] to 9.59% [397/4138]). Any changes to Sitting, Lying, and Standing classification with and without the barometer were negligible. As the barometer proved beneficial for stair classification, we included barometer data in our main analyses.

#### Subject Sample Size for Training Data

We evaluated the effect of subject sample size on AR performance to determine the dependence of classifier accuracy on the number of subjects in the training set. For each population model and the Healthy-trained gait impairment model, we varied the number of subjects used in training from two to fourteen, selecting 1200 instances at random from the available training subjects. We chose 1200 instances because this was the maximum number available between the two subjects with the least amount of data. The number of instances was kept constant, though more subjects were added to the training set to determine the effect of intra-subject variability rather than simply the addition of more data. Each instance is a 10 second clip with 90% overlap between clips, so this corresponds to about 1200 seconds (20 minutes) of data. The models were evaluated on the remaining test subjects to determine the mean recall. This process was repeated 1000 times for each subject sample size to provide the mean and 95% CIs.

### Statistical Analysis

The primary measure of model performance was mean recall. Recall refers to the percentage of correct classifications for a single activity out of all instances of that activity. Recall is the multiclass version of sensitivity, which is only defined for problems with two classes. For the population and environmental models, mean recall was computed for each model as recall averaged across activity classes. Performance of the gait impairment models was additionally evaluated based on the misclassification between stationary (Sitting, Lying, and Standing) and ambulatory (Stairs Up, Stairs Down, and Walking) activities.

Paired *t* tests were used to compare mean recall between the Healthy-to-Stroke and the Stroke-to-Stroke population models, as well as to compare mean recall between the Lab 1-to-Home and the Lab 1-to-Lab 2 environmental models. A two-sample *t* test was used to compare mean recall between the Healthy-to-Healthy and the Stroke-to-Stroke population models. For the gait impairment models, analysis of variance (ANOVA) was used to examine variations in stationary and ambulatory recall among the three impairment groups, using the Tukey honest significant difference test for multiple comparisons. Pearson correlation coefficients were also computed to determine the association between model performance and gait impairment, including mean recall of ambulatory activities and misclassification between stationary and ambulatory activities. Statistical significance levels were set to alpha=0.05, and values are presented as mean (SD).

## Results

### Distribution of Activity Classes

**Figure 2:**
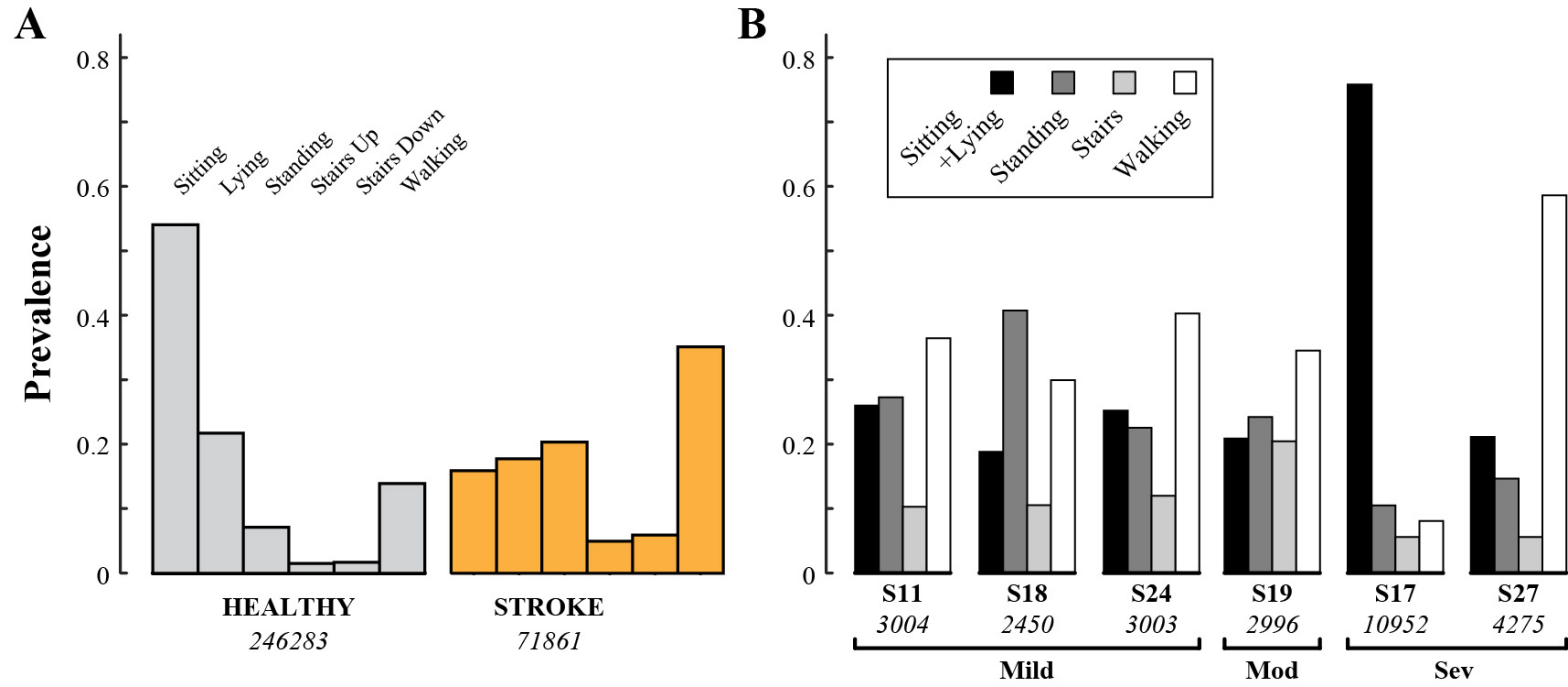
(A) Prevalence of activity classes within the healthy population (gray) and within the stroke population (orange). (B) Prevalence of classes for each of the six Stroke subjects included in the personal environmental models. The total number of instances for each population or subject is shown in italics.

The amount of data available for training and testing was affected by incidences of transmission-based data drop (poor 4G/LTE signal, leading to transmission backlog and data loss), noncompliance (subject did not label an expected activity), and mislabeling (incorrect activity selected). The percentages of activity labels affected are provided in Table 3. In conjunction with differences in the amount of time spent labeling, this produced class distributions that varied notably between subject groups (Figure 2). The Healthy population generated more than three times the number of instances than the Stroke population (246,283 vs 71,861). Stationary activities accounted for 82.88% (204,123/246,283) of the Healthy data and 53.99% (38,801/71,861) of the Stroke data. Walking was the most prevalent activity for Stroke subjects, accounting for 35.12% (25,234/71,861) of the instances for that population.

Of the six subjects included in the environmental models, three had mild gait impairment, one had moderate gait impairment, and two had severe gait impairment. The distribution of classes for each of these subjects is given in Figure 2.

**Table 3:** Data loss: average and 95% CIs for percentage of activity labels lost to transmission drop, noncompliance, and mislabeling for each population and environment.

Loss type		Healthy	Stroke
**Transmission drop**			
	Home	50.6 (40.8-60.4)	44.9 (28.9-61.4)
	Lab1	N/A^a^	11.9 (0.8-23.0)
	Lab 2	N/A	31.0 (14.2-47.9)
**Noncompliance**			
	Home	15.2 (4.9-25.4)	23.6 (13.3-33.9)
	Lab1	N/A	3.7 (0.4-7.1)
	Lab 2	N/A	1.4 (0-3.0)
**Mislabeling**			
	Home	2.5 (1.0-3.9)	12.4 (1.2-23.0)
	Lab1	N/A	12.1 (0.8-23.3)
	Lab 2	N/A	23.3 (8.9-37.7)

^a^N/A: not available.

**Figure 3:**
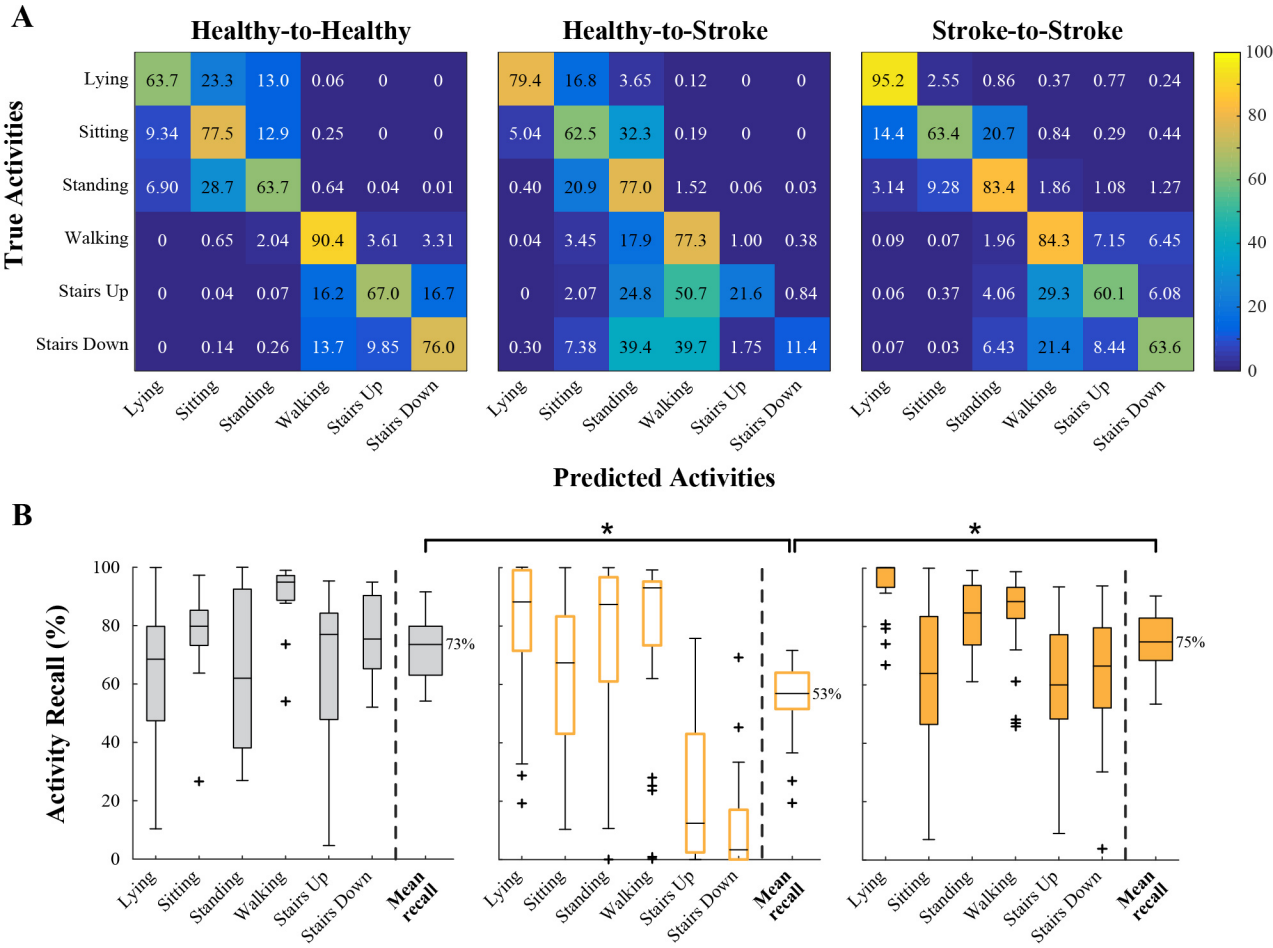
(A) Confusion matrices for each population model, showing average activity recall across test subjects. (B) Boxplots of activity recall for each population model.

### Population Models

As expected, the Stroke-to-Stroke model had higher recall across activities than the Healthy-to-Stroke model (Figure 3), showing particular improvement when classifying the Stairs activities.

The mean recalls of the Healthy-to-Healthy, Healthy-to-Stroke, and Stroke-to-Stroke models were 73% (SD 11), 53% (SD 13), and 75% (SD 10), respectively (Figure 3). The Healthy-to-Stroke model performed significantly worse than the Healthy-to-Healthy model (two-sample *t* test, *P*<.001) and the Stroke-to-Stroke model (paired *t* test, *P*<.001). There was no significant difference between the Healthy-to-Healthy and the Stroke-to-Stroke models (two-sample *t* test, *P*=.52).

Average misclassification of ambulatory activities as stationary, and vice versa, was low for the Healthy-to-Healthy (<1.1%) and the Stroke-to-Stroke models (<4.4%). For the Healthy-to-Stroke model, average misclassification of stationary activities as ambulatory was also low (0.77%, 297/38,801), but much more pronounced for ambulatory activities mistaken as stationary ones (30.88%, 10,210/33,060). That is, using a Healthy training set to test on post-stroke activities underestimated ambulation and overestimated less mobile activities such as sitting and standing.

### Gait Impairment Models

**Figure 4:**
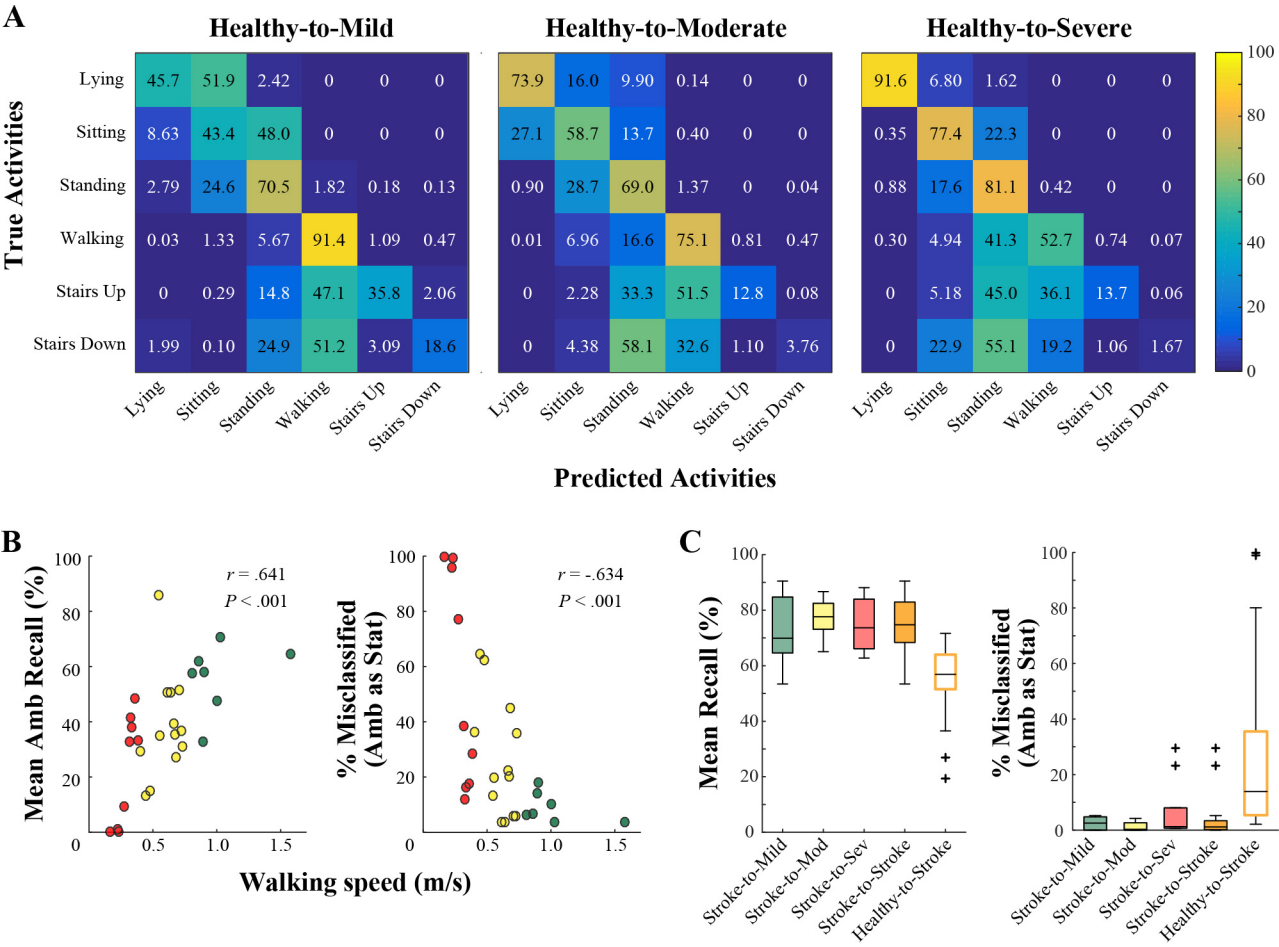
(A) Confusion matrices for each gait impairment in the Healthy-to-Stroke model. (B) For a Healthy training set, walking speed in the 10MWT is positively correlated with mean recall across ambulatory activities (left) and negatively correlated with misclassification of ambulatory activities as stationary (right) when using a Healthy training set. (C) For a Stroke training set, mean recall and misclassifications of ambulatory activities are similar between gait impairment groups.

Confusion matrices for the three gait impairment models using a Healthy training set are shown in Figure 4. For stationary activities, the mean recalls of the Healthy-to-Mild, Healthy-to-Moderate, and Healthy-to-Severe models were 59% (SD 7), 74% (SD 16), and 74% (SD 13), respectively. An ANOVA on the mean stationary recall for each stroke subject yielded no significant variation between the three models (F_2,26_=1.83, *P*=.18). For ambulatory activities, the respective mean recalls of the three models were 56% (SD 13), 35% (SD 19), and 23% (SD 20). An ANOVA on the mean ambulatory recall for each subject revealed significant variation between the three models (F_2,26_=6.96, *P*=.004).

A post-hoc Tukey test confirmed that the Healthy-to-Mild and Healthy-to-Severe mean recall was significantly different (*P*=.003); the Healthy-to-Moderate model did not have significantly different mean recall from Healthy-to-Mild (*P*=.11) or Healthy-to-Severe (*P*=.12).

In fact, across all stroke participants in this study, there was a significant, moderate correlation between mean recall in the ambulatory activities and walking speed in the 10MWT (Figure 4; *r*=.641, *P*<.001). That is, the performance of the Healthy training set to classify stairs and walking activity declined as gait impairment increased. On the other hand, there was no association between mean recall in stationary activities and walking speed (*r*=−.262, *P*=.17).

The effect of gait impairment appeared most pronounced in the classification of ambulatory activities. Average misclassification of stationary activities decreased slightly with gait impairment, from 1.07% (97/9075) in Mild to 0.12% (19/16,077) in Severe. Average misclassification of ambulatory activities as stationary activities increased substantially with gait impairment, from 10.21% (818/8014) in Mild to 30.02% (3354/11,172) in Moderate, and to 51.01% (7077/13,874) in the Severe subjects. The Healthy-to-Mild model more accurately distinguished between stationary and ambulatory activities, with mean recalls of 99% (SD 1) and 90% (SD 5), respectively.

There was a moderate negative correlation between misclassification of ambulatory activities and walking speed in the 10MWT (Figure 4; *r*=−.634, *P*<.001). There was no association between misclassification of stationary activities and walking speed (*r*=−.307, *P*=.11). Thus, gait impairment hindered the classification of ambulatory activities, without impacting stationary activities.

The mixed-impairment training data from the Stroke-to-Stroke model recognized activities more accurately than the Healthy-to-Stroke model, across impairment levels. The mean recalls of the Stroke-to-Mild, Stroke-to-Moderate, and Stroke-to-Severe models were 72% (SD 16), 78% (SD 11), and 74% (SD 16), respectively (Figure 4). An ANOVA on the mean recall for each subject yielded no significant variation between the three models (F_2,26_=1.33, *P*=.28).

Average misclassification of stationary activities was similar across gait impairment groups for the Stroke-to-Stroke model, with 2.7% of instances misclassified in Mild, 3.7% in Moderate, and 1.8% in Severe (Figure 4). Average misclassification of ambulatory activities as stationary activities occurred more frequently for persons with severe gait impairment, with 6.9% of instances misclassified in Severe versus 2.4% in Mild and 1.3% in Moderate. Most of these errors in the Severe subjects resulted from confusing stairs activity with standing, presumably due to the slower speeds and longer pauses on the stairs.

### Environmental Models

**Figure 5:**
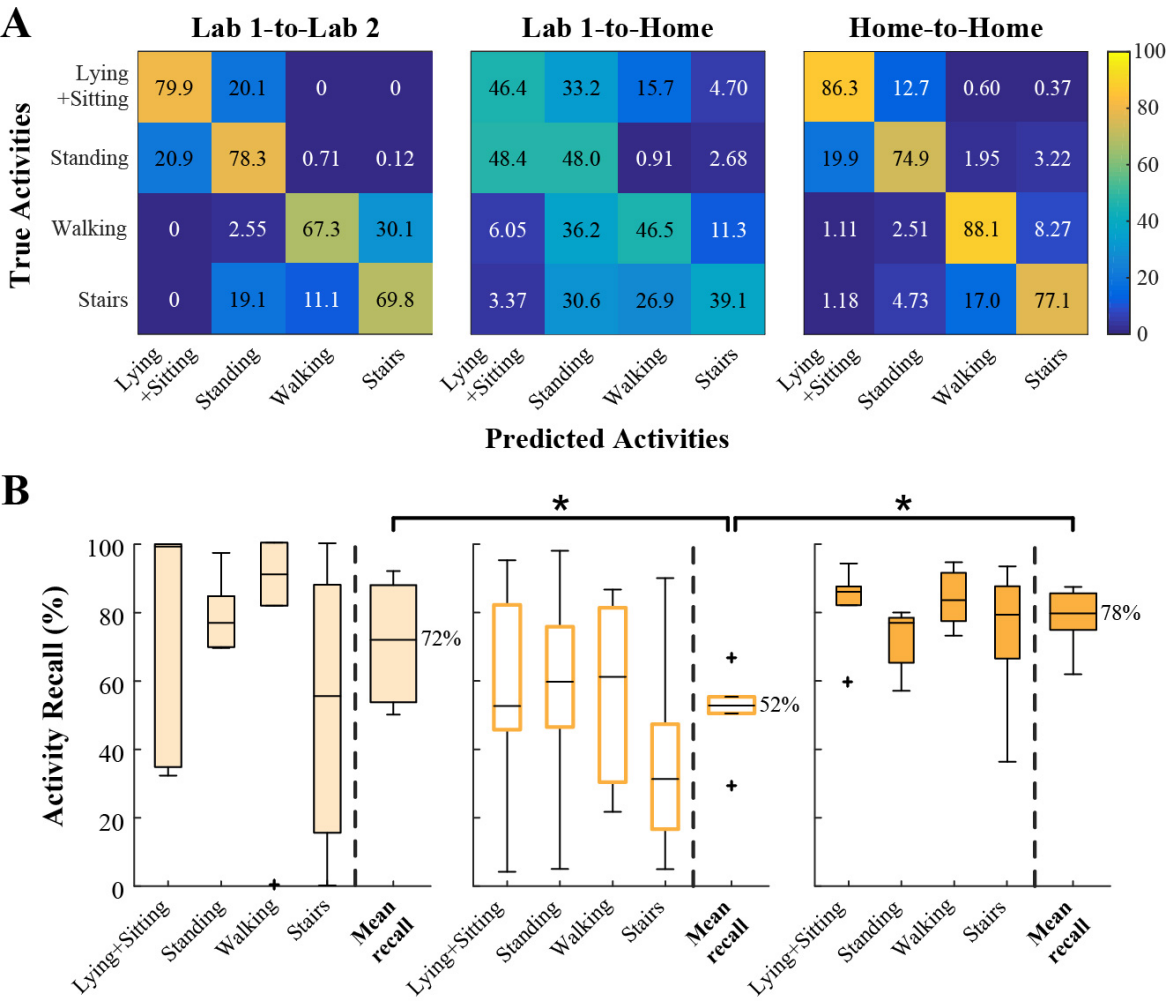
(A) Confusion matrices and (B) boxplots of activity recall for the personal environmental models.

The mean recall values of the Lab 1-to-Lab 2, Lab 1-to-Home, and Home-to-Home models were 72% (SD 18), 52% (SD 12), and 67% (SD 7), respectively (Figure 5). The Lab 1-to-Home model performed significantly worse than then Lab 1-to-Lab 2 model (*P*=.024) and the Home-to-Home model (*P*<.001). There was no significant difference between the Lab 1-to-Lab 2 and the Home-to-Home models (*P*=.43). In the Lab 1-to-Home model, average misclassification of stationary activities as ambulatory occurred in 17.86% (2,075/11,618) of instances, compared with 0.54% (7/1,296) in the Lab 1-to-Lab 2 model and 1.58% (179/11,316) in the Home-to-Home model. More drastically, the Lab 1-to-Home model misclassified ambulatory activities as stationary on an average of 40.34% (2,273/5,635), compared with 6.57% (68/1,035) for the Lab 1-to-Lab 2 model and 4.10% (218/5,320) for the Home-to-Home model.

To compare the effects of environment in a practical AR implementation, we also examined global models for Lab 1-to-Lab 2, Lab 1-to-Home, and Home-to-Home. The mean recall values of these models were 80% (SD 11), 65% (SD 14), and 61% (SD 9), respectively. There was no significant difference between the Lab 1-to-Lab 2 and the Lab 1-to-Home global models (*P*=.07), nor between the Lab 1-to-Home and the Home-to-Home global models (*P*=.53). The Home-to-Home global model performed significantly worse than the Lab 1-to-Lab 2 global model (*P*=.004). In the Lab 1-to-Home global model, average misclassification of stationary activities as ambulatory occurred in 0.84% (98/11,618) of instances, compared with 0.54% (7/1,296) in the Lab 1-to-Lab 2 global model and 1.34% (156/11,618) in the Home-to-Home global model. More drastically, the Lab 1-to-Home model misclassified ambulatory activities as stationary on an average of 12.49% (704/5,635) of the time, compared with 3.57% (37/1,035) for the Lab 1-to-Lab 2 model and 3.07% (173/5,635) for the Home-to-Home model.

In summary, personal models exhibited a marked improvement in mean recall when using a Home training set rather than a Lab training set to classify Home activities. Global models that were run on six subjects did not show such improvement. Both types of AR models showed similar trends in reduced misclassification of Home ambulatory activities when using a Home rather than Lab training set.

### Sample Size of Training Subjects Achieves Near-Optimal Performance

**Figure 6:**
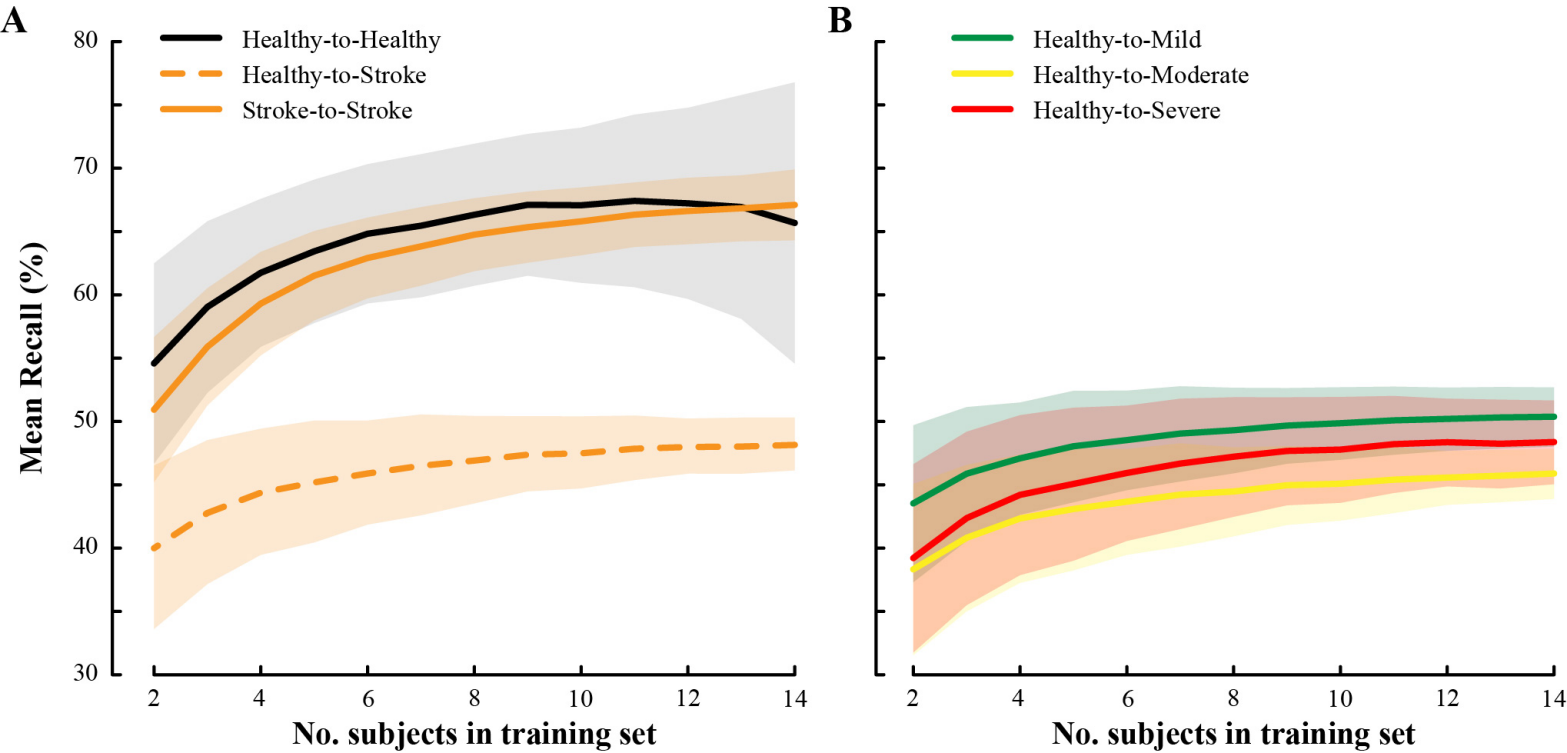
Average and SD of mean recall for (A) population models and (B) gait impairment models using different subject sample sizes in training data (1200 random instances pulled, repeated 1000 times).

Mean recall increased with training set sample size in our population models (Figure 6). The Healthy-to-Healthy model performance plateaued after about 10 training subjects. The Healthy-to-Stroke performance plateaued at about 12 subjects, regardless of the impairment severity group used for testing (Figure 6). The Stroke-to-Stroke model began to plateau at 14 subjects. Therefore, we recommend using a training pool of at least 14 subjects for optimal AR performance for persons with stroke. More subjects may be necessary for AR involving more activity classes than the six used in this study.

This suggests that our models, using 14 healthy and 29 stroke subjects in training, achieved near-optimal performance. Note that the analyses presented in the main text included all available instances for each subject. Including more training instances would produce higher mean recall than those given for our population models. For a larger number of instances, fewer subjects may be necessary to achieve the point of marginal performance gains.

Healthy training data yielded only marginal improvements using more than 10-12 subjects. Stroke training data yielded continued improvements using up to 14 subjects.

## Discussion

### Principal Findings

We have shown that training AR models on a mixed-impairment stroke population improves activity recognition for persons with stroke compared with training on a healthy population, increasing mean recall from 53% to 75%. Models trained on either the healthy population or patients with mild gait impairment performed poorly when classifying ambulatory activities in patients with severe gait impairment, lowering mean recall to 23% by increasingly misclassifying ambulatory activities as stationary ones.

Finally, personal models trained on in-lab activities performed poorly when tested on at-home activities, with 56% recall. Global models, which account for inter-subject variability, may be less susceptible to variability in activities between environmental contexts. However, the effect of environment on global models remains to be demonstrated with a sufficient number of subjects in the training set; using only six subjects in the environmental global model analysis is likely insufficient to achieve optimal model performance. Taken together, our results suggest that future community monitoring systems for persons with stroke should incorporate activity training data collected outside the laboratory using cohorts with similar gait impairment or a wide range of gait impairments.

### Comparison With Prior Work

Our results for the stroke population align with previous work validating AR reliability for persons with neurological injury when using training data from a young, healthy cohort [14-16,18]. We have extended this analysis to stroke by comparing AR across levels of gait impairment, finding that the healthy-trained model increasingly underestimated ambulation with impairment severity. This is especially problematic as it affects a population that already exhibits reduced ambulation. Conversely, misclassification was reduced across impairment groups using a model trained on a mixed-impairment stroke cohort (Figure 4). Our results indicate that training sets incorporating a broad range of gait impairments may be generally sufficient to classify the activities of a stroke subject.

Our study also offers several contributions to community monitoring research for persons with stroke. We have presented an activity recognition system using smartphone technology that allows users to independently label activity data outside the laboratory. Using this system, we have evaluated the validity of various activity recognition models when classifying activities in persons with stroke. Our findings agree with a recent study by Albert et al [25], in which support vector machine models trained on home activity data outperformed models trained on lab data when classifying mobility-related activities for patients with incomplete spinal cord injury. Our results further demonstrate the need to train AR models using data representative of home activities as best as possible for the population of interest. We expect this would also hold for activity data collected from other movement sensors, such as inertial measurement units (IMUs).

### Limitations

While this work is an important first step in highlighting potential issues in AR for community-based monitoring, our findings should be considered in the context of several limitations. For one, the healthy cohort was not age-matched to the stroke cohort, which may underestimate the performance of the Healthy-to-Stroke population model. Nevertheless, patients with stroke have reduced walking speeds relative to age-matched controls [26], and we do not expect that age-matching would negate our overall findings in the population models. Indeed, age and neurological injury both appear to negatively impact AR performance when training with activities from a young, healthy population.

The positioning of the mobile phone may present another limitation. Other studies using mobile phone data have placed the phone in the pocket and achieved better resolution of stationary activities than the models presented here. For example, Kwapisz et al [27] achieved high sensitivities for both sitting and standing (>93%) with a mobile phone placed in the pocket. Sensor data collected from the waist may not be adequate to distinguish between sitting and standing postures. We decided to keep the phone in a belted pouch at the waist to minimize discomfort, avoid the need for large-pocketed clothing, and maintain consistent phone access for all subjects during labeling.

A third limitation of our study was the reduced amount of home data available for AR validation in the stroke population, resulting from data loss and protocol design. Most of the data loss resulted from transmission-based data drops, which happened in cases of poor 4G/LTE signal. Specifically, continuously poor signal would lead to a data backlog and prevent new data from being captured. This was a problem with the CIMON app that has since been improved for future studies. Furthermore, because stroke subjects only completed one session of at-home labeling, we expect the Home-to-Home personal models to have slightly overestimated performance, though this is likely negligible relative to the poor performance of the Lab-to-Home models. Follow-up studies should collect activity data on different days and in more varied community environments to better capture variance in behavior.

Future work should consider the amount of training data needed for satisfactory AR performance in a stroke cohort. Model accuracy changes with the sample size of healthy and stroke subjects included in the training set. We found that about 10 healthy subjects and 14 stroke subjects achieved satisfactory AR accuracy, using 20 minutes of total activity data for model training (Figure 6). However, the amount of training data is not the sole determinant of model quality. As we have demonstrated in this study, consideration of target population and environment are crucial to maximize AR performance.

### Conclusions

Improving the reliability of AR algorithms for persons with stroke has significant benefits for home and community monitoring. Wearable technology paired with AR will allow clinicians to construct and supervise remote/home rehabilitation programs, utilizing data-driven feedback about patient activities. Our recommendation for future stroke-based AR models is to use training data from a balanced distribution of gait impairment levels, thereby including as much variety as possible to improve performance across all levels. Future studies should further examine real-world activity labeling to improve the ability of AR models to generalize across multiple environmental contexts, not just in the laboratory and at home. These findings may help guide the construction of future AR models for persons with stroke and other clinical populations.

## Abbreviations

10MWT: 10-meter walk test
AR: activity recognition
HIPAA: Health Insurance Portability and Accountability Act of 1996
RF: random forest
RUS: random undersampling
SD: standard deviation
